# Digital pen technology for conducting cognitive assessments: a cross-over study with older adults

**DOI:** 10.1007/s00426-020-01452-8

**Published:** 2020-12-17

**Authors:** A. Heimann-Steinert, A. Latendorf, A. Prange, D. Sonntag, U. Müller-Werdan

**Affiliations:** 1 Charité – Universitätsmedizin Berlin, corporate member of Freie Universität Berlin, Humboldt-Universität zu Berlin, and Berlin Institute of Health; Geriatrics Research Group, Reinickendorfer Str. 61, 13347 Berlin, Germany; 2 German Research Center for Artificial Intelligence (DFKI GmbH), Stuhlsatzenhausweg 3, 66123 Saarbruecken, Germany

## Abstract

Many digitalized cognitive assessments exist to increase reliability, standardization, and objectivity. Particularly in older adults, the performance of digitized cognitive assessments can lead to poorer test results if they are unfamiliar with the computer, mouse, keyboard, or touch screen. In a cross-over design study, 40 older adults (age M = 74.4 ± 4.1 years) conducted the Trail Making Test A and B with a digital pen (digital pen tests, DPT) and a regular pencil (pencil tests, PT) to identify differences in performance. Furthermore, the tests conducted with a digital pen were analyzed manually (manual results, MR) and electronically (electronic results, ER) by an automized system algorithm to determine the possibilities of digital pen evaluation. ICC(2,k) showed a good level of agreement for TMT A (ICC(2,k) = 0.668) and TMT B (ICC(2,k) = 0.734) between PT and DPT. When comparing MR and ER, ICC(2,k) showed an excellent level of agreement in TMT A (ICC(2,k) = 0.999) and TMT B (ICC(2,k) = 0.994). The frequency of pen lifting correlates significantly with the execution time in TMT A (*r* = 0.372, *p* = 0.030) and TMT B (*r* = 0.567, *p* < 0.001). A digital pen can be used to perform the Trail Making Test, as it has been shown that there is no difference in the results due to the type of pen used. With a digital pen, the advantages of digitized testing can be used without having to accept the disadvantages.

## Introduction and overview

In geriatrics, neuropsychological assessments are used to measure cognitive abilities and to detect changes in cognitive functioning (Tuokko & Hadjistavropoulos, 1998). There is a wide range of cognitive assessments testing neurobehavioral disorders in memory, language, emotions, attention, perception, executive functions, or visuospatial skills (Minagar, Finney, & Heimann, 2015). With advancing digitalization, the possibilities for digitalized cognitive assessments using a computer or tablet have expanded. In particular for the early detection of changes in the elderly and in patients with mild cognitive impairment, many digitalized assessments exist (Wild, Howieson, Webbe, Seelye, & Kaye, 2008; Woo, 2008). The advantages of digitalized assessments are described extensively in the literature. These advantages include an increase in reliability, objectivity, and standardization (Sternin, Burns, & Owen, 2019). An automated administration, scoring and interpretation of data, and the possibility of a convenient data storage are further advantages of digitalized assessments (Cernich, Brennana, Barker, & Bleiberg, 2007; Sternin et al., 2019). Furthermore, tests are able to measure additional data such as response rates on the millisecond level, thereby providing more detailed insight into the patients capabilities. Less is said about the disadvantages of digitalized cognitive assessments. Some literature findings stated that clinicians should use tests on a computer or a tablet with caution and with consideration of potential technical complications (Bracken, Mazur-Mosiewicz, & Glazek, 2019; Cernich et al., 2007). Cernich et al. focus primarily on the possible technical problems in hardware, software, peripherals, the display, connections, and bandwidth as well as program considerations (Cernich et al., 2007). However, there are not only the technical challenges; especially for older adults, the computer with mouse, keyboard, and number pad or possibly a touchscreen can be intimidating or unfamiliar in the beginning. Therefore, initial training is necessary (Fortuny & Heaton, 1996). A study by Weber et al. showed that low acceptance of digitalized assessments and patients with a more negative attitude toward computers, correlates with poorer test results in attention tasks (Weber, Fritze, Schneider, Kuhner, & Maurer, 2002). Visual impairment or age-related vision loss as well as cognitive impairment or motor impairment may also cause relevant problems that are not related to the test results (Silverberg et al., 2011). Bauer et al. also stated that the results can be falsified if the patient has to use their non-dominant hand to manipulate a mouse or a touchscreen, e.g., in hemiparetic patients. They conclude that it cannot be assumed that the results of a paper–pencil test are equal to computerized test results (Bauer et al., 2012). Since computerized assessments showed only moderate correlations with paper–pencil tests (Silverberg et al., 2011), new norm values for results classification have to be collected. The generation of new norm values is associated with high effort and extensive costs.

To benefit from the advantages of digitalization in cognitive testing (e.g., automatic soring, additional information) without having to accept the disadvantages (e.g., unfamiliarity), digital tools besides computer, tablet, mouse, and touchscreen can be a solution. A digital pen allows digitalizing all notes written with the pen on normal paper (more information in section Methodology). The positive effects of digital pen technology were already investigated in learning (Boyle & Joyce, 2019) and as a method to detect conducted homework (Rawson, Stahovich, & Mayer, 2017). Within the study by Rawson et al., a digital pen was used to automatically and reliably record the homework activity to find a connection with academic achievement (Rawson et al., 2017). Further concepts describe the possibilities of digital pen usage as an intuitive assistance tool for persons with dementia to improve communication, for example, when writing or answering emails (Prange, Sandrala, Weber, & Sonntag, 2015). Within the demo paper of Prange et al., a digital pen was used that streams its data via Bluetooth directly to a server, while the dementia patients write with a seemingly normal pen on paper with an invisible dot pattern. The authors of the demo paper also point out that the cognitive status of a participant can affect pen holding and (fine-) motor movements (Prange et al., 2015).

The possible advantages of digitalized cognitive assessments (Cernich et al., 2007; Sternin et al., 2019) and the advantages of a digital pen (Boyle & Joyce, 2019; Rawson et al., 2017) have already been examined in various studies. The use of a digital pen in cognitive testing can help to take advantage of digital cognitive assessments without the disadvantages of unfamiliarity or lack of acceptance. The aim of the presented study was to show whether the digital pen technology could be used to conduct cognitive assessments with older people, in contrast to regular paper–pencil execution. Therefore three hypotheses were generated:

H1: The execution time in TMT A and TMT B is not influenced by the type of pen (duration DPT = duration PT).

H2: The electronically measured execution time (ER) correlates significantly with the manually measured execution time (MR).

H3: The additional, electronically measured parameters (number of pen lifts, errors, omitted circles, all circles hit, correct order) correlate significantly with the execution time.

## Methodology

### Study design and process

**Fig. 1:**
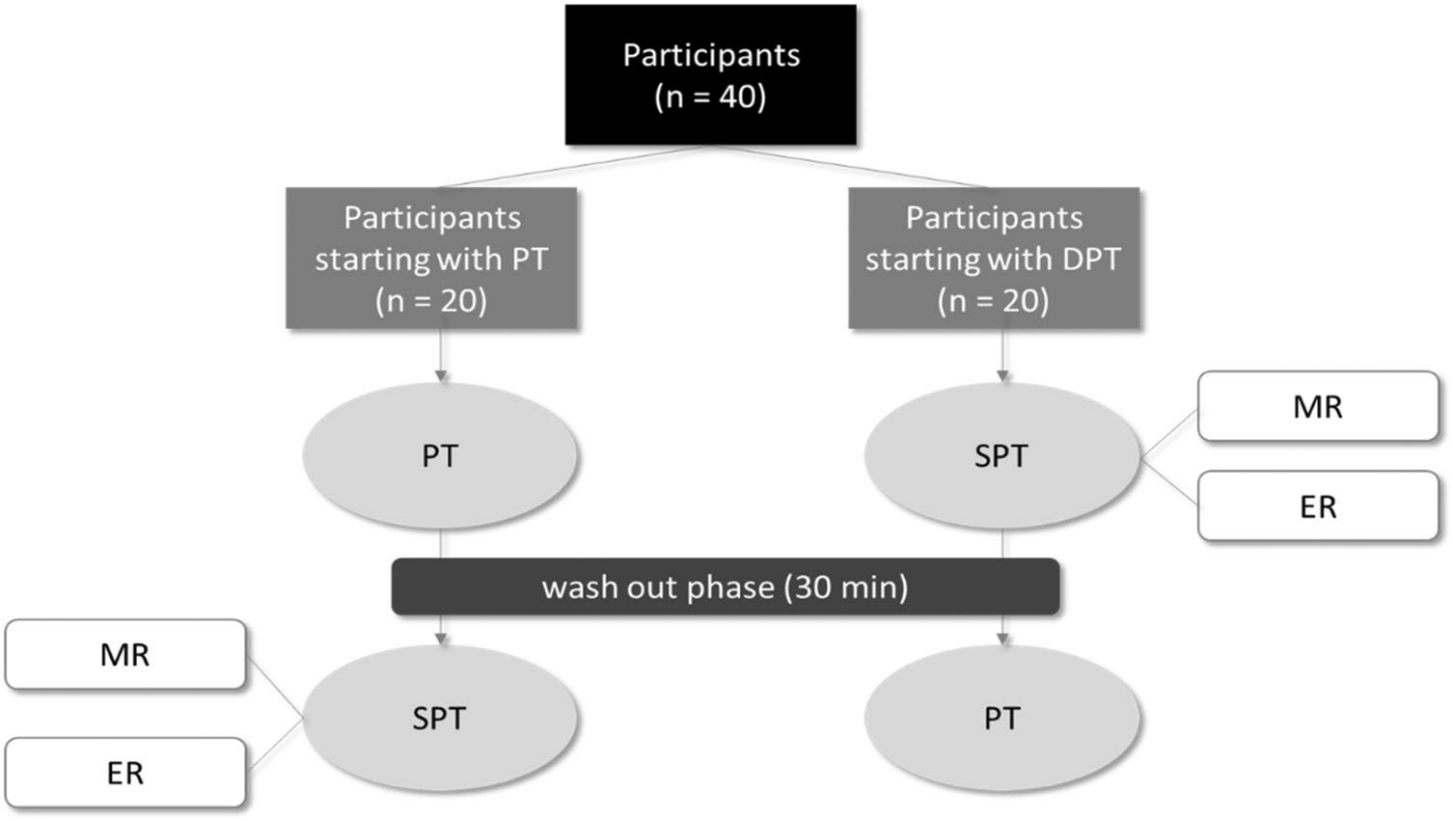
Procedure of the cross-over design study. *PT* pencil test, *DPT* digital pen tests, *MR* manual results, *ER* electronic results

To test the hypotheses, 40 participants were included in the presented cross-over design study. Inclusion criteria for participants were a minimum age of 65 and a participants’ signed informed consent. Exclusion criteria were severe cognitive disorders, mental diseases, severe auditive, visual, linguistic, sensory or motor limitations, chronic pain, or a legal representative. The participants conducted the Trail Making Test A (TMT A) and the Trail Making Test B (TMT B) (Reitan, 1992) as one of the most widespread assessments for the general examination of brain function (Tischler & Petermann, 2010). In the presented study, we used the TMT version of the CERAD (Consortium to Establish a Registry for Alzheimer’s Disease). In TMT A and B, participants had to connect numbers (TMT A) or numbers and letters alternating (TMT B), respectively, in ascending order, without lifting the pen from the paper. The required amount of time (execution time) represents the test results in TMT A and B. If the participant made a mistake, it was immediately corrected by the study personnel, by pointing to the error and the solution, which is done very quick and without pausing the time. Therefore, the number of errors affect the participant’s score only in that the correction of errors is included in the completion time for the task. The number of errors was not documented separately. Participants used their dominant hand for the execution. All participants conducted the cognitive tests twice: once with a pencil (pencil tests, PT) and once with a digital pen (digital pen tests, DPT), in both cases on paper. Therefore, the surface on which the test was performed did not affect the test results. Trained study personnel gave the instruction in PT and DPT. The execution time in DPT was measured by the study personnel (manual result, MR) and by an automized system algorithm (electronic result, ER). To decrease sequence effects, half of the participants started with a pencil the other half with the digital pen (sex-stratified, Fig. 1). There was a wash out phase between PT and DPT of approx. 30 min. During this phase, participants completed a self-developed questionnaire to collect socio-demographic data and a questionnaire to record the technology commitment (Neyer, Felber, & Gebhardt, 2012). The self-developed questionnaire for collecting socio-demographic data included questions on age, marital status, education, income, and health status (Table 1). The questionnaire for recording technology commitment comprises 12 statements. Four statements each relate to the acceptance of technology (e.g., I am very curious about new technical developments), technology competence (e.g. In dealing with modern technology I am often afraid to fail), and technology control (Whether I am successful in the application of modern technology depends to a large extent on me). The participants rated their agreement with each statement on a 5-point likert scale (do not agree at all–agree completely) (Neyer et al., 2012).

**Table 1:** Socio-demographic data and technology commitment of the sample

Variable	*N* = 40
Age [Ø years]	74.4
Gender [%]	
Male	50.0
Female	50.0
Education [%]	
Low-level education	2.5
Mid-level education	40.0
High-level education	57.5
Marital status [%]	
Single	10.0
Married	57.5
Divorced	15.0
Widowed	17.5
Income per month [%]	
< € 1500	25.0
€ 1501–2500	40.0
€ 2501–3500	7.5
> € 3500	22.5
Prefer not to say	5.0
Subjective health [%]	
Rather/very good	62.5
Moderate	35.0
Rather/very poor	2.5
Technology commitment [points]	
Score [12–60]	45.2
Subscore acceptance [4–20]	13.5
Subscore competence [4–20]	16.0
Subscore control [4–20]	15.6

### Digital pen technology

**Fig. 2:**
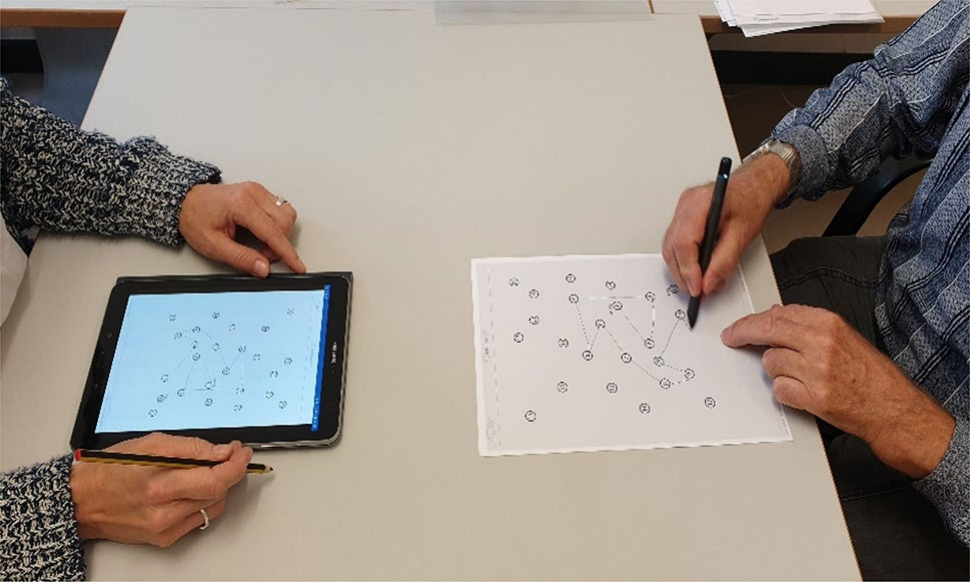
A participant (right) connects the numbers of the TMT A with a digital pen. The process can be seen live by the researcher (left)

As a digital pen, participants used the Neo SmartPen N2 (https://www.neosmartpen.com). Weighing 22 g, the pen is slightly heavier and thicker than a usual pencil. A small infrared camera within the pen, which recognizes the unique pattern of dots printed on each page, captured all written or drawn information. This enables a direct digitalization of user input in real time (Fig. 2). Accordingly, in preparation of this study, the blank forms for the TMT A and TMT B were printed on paper with the almost invisible dot pattern.

**Fig. 3:**
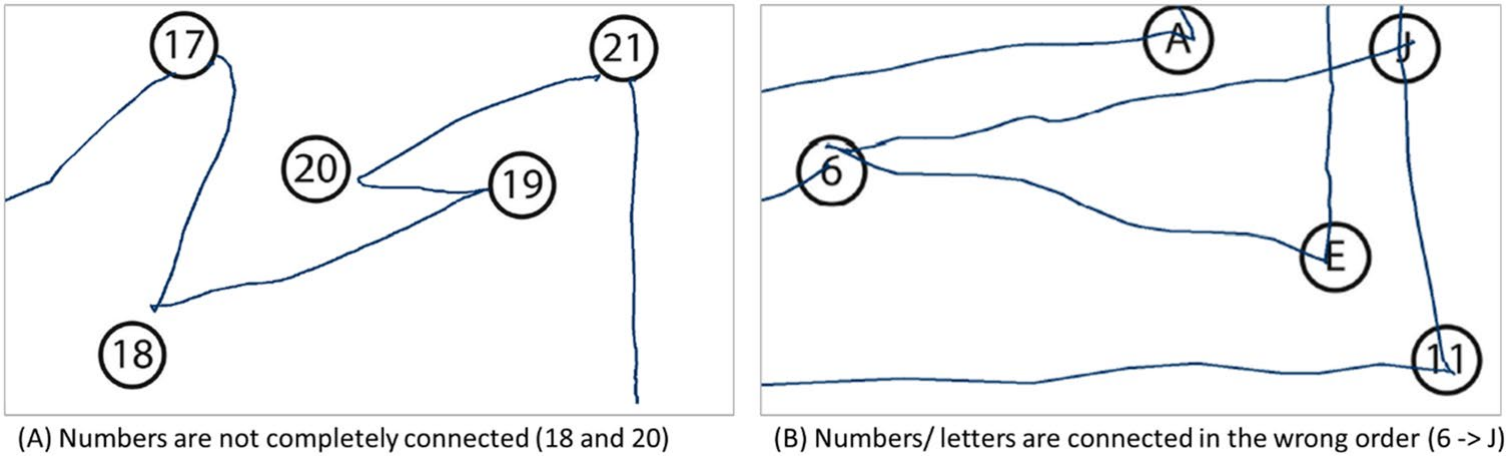
Possible errors detected by the electronic evaluation

Within the publicly funded project, InteraKT [Interactive cognitive assessment tool, www.intera-kt.de, (Sonntag, 2017)] for the electronic evaluation of the test results was developed. The digital pen streams the recorded ink strokes via Bluetooth on-the-fly to the tablet application, which in turn forwards the data to a backend server for further automatic analysis. Ink strokes are treated as a series of timestamped x/y coordinates, from which we calculate the execution time (the time difference between the first stroke and the last stroke). Having a digital representation of the printed paper allows us to synchronize the participant's input with the correct locations of the numbered nodes of the test. This way, we are not only able to analyze the execution time, but also the connections drawn between numbers during the assessment. In contrast to the manual test, the electronic test allows other parameters to be recorded in addition to the execution time. For example, the automatic evaluation indicates how often the pen was lifted from the paper when connecting the numbers or how many errors were made. Errors were counted if, for example, a number or letter was not completely connected or the numbers/letters were connected in the wrong order (Fig. 3).

### Data analysis

All data and test results were analyzed using SPSS. The PT and DPT results and the MR and ER were compared using paired samples *t* test, Pearson correlation, and intraclass correlation (ICCs(2,k); two-way random effects, absolute agreement). According to Cohen et al., Pearson correlation coefficients higher than 0.10 indicate weak correlation, values higher than 0.30 indicate moderate correlation and values higher than 0.50 indicate high linear correlation (Cohen, 1988). According to Cicchetti et al., ICC values less than 0.40, between 0.40 and 0.59, between 0.60 and 0.74, and values higher than 0.75 indicate poor, fair, good, or excellent reliability, respectively (Cicchetti, 1994).

## Results

### Sample

Forty older adults (age M = 74.4 ± 4.1 years, range 67–85 years) were included in the cross-over design study. Half of them were female. Most of the participants were well-educated (57.5% high-level education), married (57.5%), and right-handed (95.0%). There were no statistical differences between participants who performed PT first (*n* = 20, age: 74.4 ± 3.7 years, 50% female) and the participants who performed DPT first (*n* = 20, age: 74.3 ± 4.6 years, 50% female) regarding socio-demographic data and technology commitment. Differences were only seen in income (*t*(39) = − 2.014, *p* = 0.05).

### Comparison of performance in pencil and digital pen test

Table 2 reports the mean values and standard deviation of PT and DPT for TMT A and TMT B, the differences, and the 95% confidence intervals of average differences. *T* test showed no significant differences between PT performance and DPT performance for TMT A (*t*(39) = − 1.71, *p* = 0.095) and TMT B (*t*(39) = − 1.19, *p* = 0.243). Pearson correlations showed a moderate positive correlation for TMT A (*r* = 0.432, *p* = 0.005) and strong positive correlation for TMT B (*r*(38) = 0.651, *p *= 0.000). ICC(2,k) showed a good level of agreement for TMT A (ICC(2,k) = 0.668) and TMT B (ICC(2,k) = 0.734, Table 2).

**Table 2:** Comparison of performance in PT and DPT

	PT	DPT	PT–DPT			
	Mean (SD)	Mean (SD)	Diff [95% CI]	*t* test *p* value	Pearson Corr	ICC(2,k)
TMT A [in s]	36.15 (10.45)	40.96 (19.61)	4.82 [− 0.88; 10.51]	0.095	0.432^a^	0.668
TMT B [in s]	77.91 (26.05)	83.94 (42.34)	6.03 [− 4.26; 16.31]	0.243	0.651^a^	0.734

*PT* pencil tests, *DPT *digital pen tests, *SD* standard deviation, *Diff [95% CI]* differences in mean values with confidence intervals lower and upper bound, *ICC *intraclass correlation coefficient

^a^Correlation is significant at the 0.01 level (two-tailed)

Within the group of subjects who first performed the tests with a pencil, strong positive correlations between PT and DPT were found for TMT A (*r*(18) = 0.527, *p* = 0.017) and TMT B (*r*(18) = 0.915, *p* = 0.000). Within the group of subjects who first performed the tests with a digital pen, strong positive correlations between PT and DPT were found for TMT A (*r*(18) = 0.627, *p* = 0.003) and TMT B (*r*(18) = 0.783, *p* = 0.000).

**Fig. 4:**
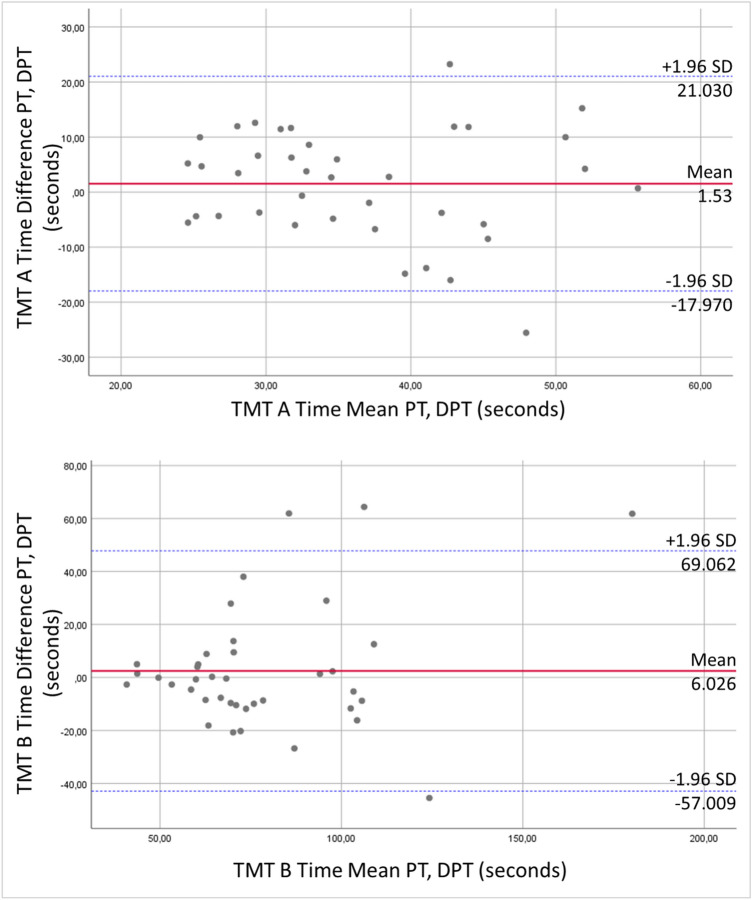
Bland–Altman plot of differences between PT and DPT vs. mean values with the presentation of level of agreement for TMT A and TMT B

The fundamental agreement in results of PT and DPT for TMT A and TMT B are also shown in the Bland Altman Plots where the differences of DPT and PT are plotted against the mean of the two measurements (Fig. 4). Two (5.0%, TMT A) and four (10.0%, TMT B) data points are outside the limits of agreement.

In addition, participants were asked whether they believed that the type of pen influenced the test results. Almost all participants (95.0%) answered the question in the negative.

### Comparison of the manual and electronic results in DPT

Table 3 reports the mean values and standard deviation of MR and ER in DPT, the differences, and the 95% confidence intervals of average differences. *T* tests showed significant differences between MR and ER for both tests. Pearson correlations showed strong positive correlations for TMT A (*r*(38) = 0.999, *p* = 0.000) and TMT B (*r*(38) = 0.999, *p* = 0.000). The ICC(2,k) demonstrated an excellent level of agreement in TMT A and TMT B (Table 3).

**Table 3:** Comparison of manual results and electronic results in DPT

	MR DPT	ER DPT	MR–ER			
	Mean (SD)	Mean (SD)	Diff [95% CI]	*t* test *p* value	Pearson corr	ICC(2,k)
TMT A [in s]	40.96 (19.61)	41.50 (19.78)	0.54 [0.32; 0.77]	0.000	0.999^a^	0.999
TMT B [in s]	83.94 (42.34)	85.21 (42.60)	1.27 [− 0.80; 3.34]	0.000	0.999^a^	0.999

*ME* manual evaluation, *AE *automatic evaluation, *SD* standard deviation, *Diff [95% CI]* differences in mean values with confidence intervals lower and upper bound, *ICC* intraclass correlation coefficient

^a^Correlation is significant at the 0.01 level (two-tailed)

**Fig. 5:**
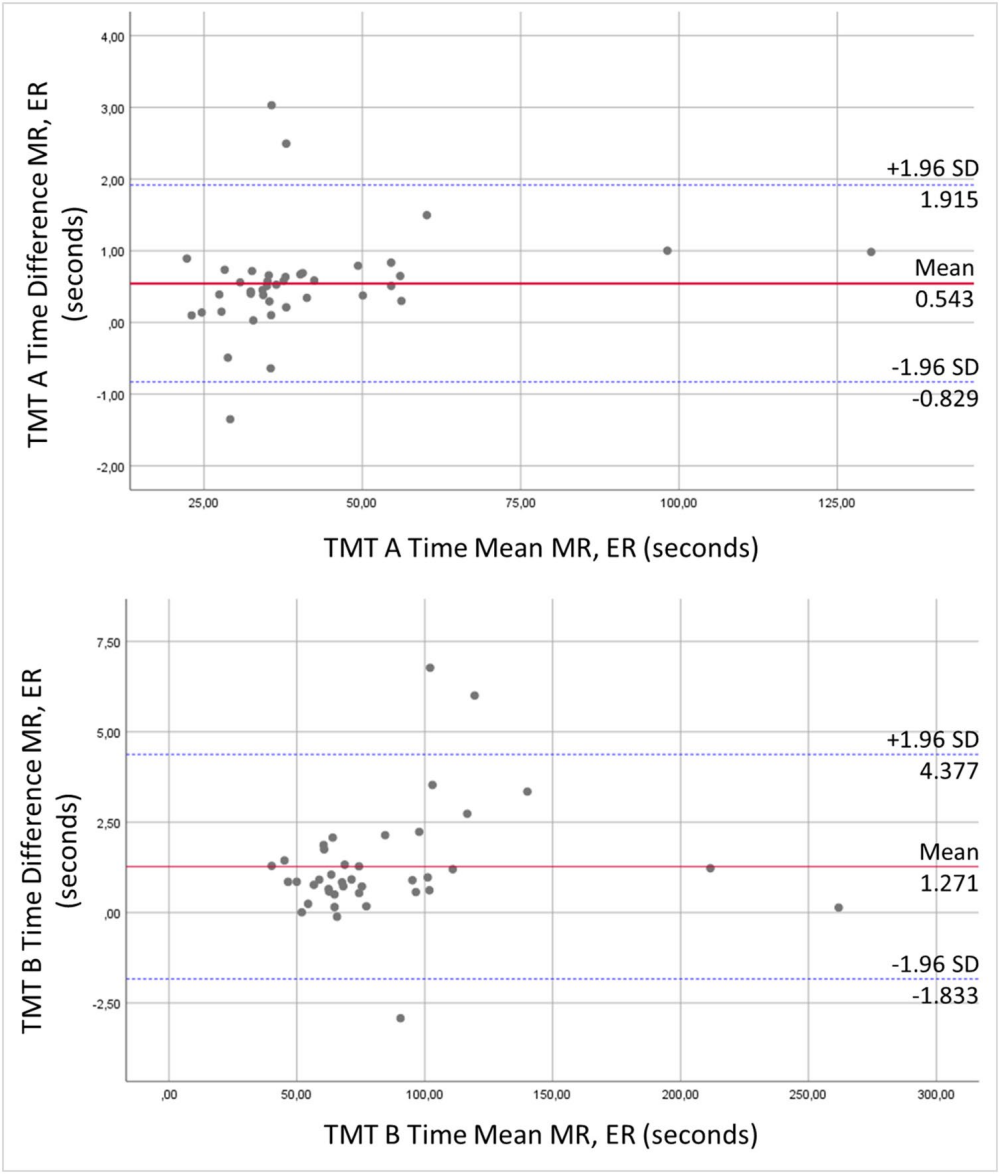
Bland–Altman plot of differences between MR and ER vs. mean values with the presentation of level of agreement for TMT A and TMT B

The Bland–Altman plot (Fig. 5) shows that three data points (7.0%) each in TMT A and TMT B are outside of the agreement limits.

### Additional parameters of ER

The algorithm was able to analyze additional parameters in test execution (e.g., pen lifts and errors). Despite the instruction not to lift the pen from the paper during the test, this was done on average 4 (TMT A) and 6 times (TMT B). The frequency of pen lifting correlates significantly with the execution time in TMT B (*r*(37) = 0.561, *p* = 0.000). The more often participants lifted the pen from the paper when connecting the circles, the more time was needed. The number of (automatically detected) errors correlates significantly with the execution time in TMT B (*r*(37) = 0.336, *p* = 0.036), but not in TMT A (*r*(37) = 0.289, *p* = 0.074). The number of omitted circles showed no significant influence on the processing time. Furthermore, there were no mean value differences in the execution time between participants who hit all circles or who did not (Fig. 3a, TMT A: *t*(37) = − 0.799, *p* = 0.430; TMT B: *t*(18,8) = − 1.242, p = 0.229) and also between participants, who connected all circles in the right order or not (Fig. 3b, TMT A: *t*(37) = 0.578, *p* = 0.567; TMT B: *t*(37) = 1.293, *p* = 0.204; Table 4).

**Table 4:** Mean values, standard deviation, and range for additional parameters in ER

	TMT A	Min–Max	TMT B	Min–Max
Pen lifted [Ø number ± SD]	4.26 ± 4.61	1–22	6.18 ± 6.88	1–33
Errors [Ø number ± SD]	2.95 ± 2.46	0–10	3.18 ± 2.42	0–9
omitted circles [Ø number ± SD]	4.64 ± 4.82	0–21	4.03 ± 4.60	0–18
All circles hit [% yes]	27.5		42.5	
Correct order [% yes]	17.5		20.0	

*N* = 39, one participant is missing in ER

## Discussion

The present study investigated the influence of the pen on the test results of older participants in TMT A and B, as well as the potential of electronic evaluation, to answer the question whether a digital pen can be used for conducting the Trail Making Test.

The first hypothesis, that the execution time is not influenced by the type of pen, is confirmed by the present study. The study shows no significant differences and good agreement in test results between PT and DPT. Even though the instruction for TMT states that a pencil should be used for TMT performance and the digital pen is heavier and thicker than a pencil, the test results were almost the same. The influence of pen design on drawing and writing, not on cognitive test performance was investigated in a study by Goonetilleke et al. The authors showed that speed and writing ability were not influenced by pen shape or pen size (Goonetilleke, Hoffmann, & Luximon, 2009). That result is additionally confirmed by the subjective assessment of the test persons that they do not believe the test result is influenced by the type of pen. Minor differences were found in the time taken for a drawing, which increases when the pen size decreased. Since the size of the pencil and the digital pen used in the present study were nearly identical, this difference could not be demonstrated in the results. The findings by Silverberg that sensory or cognitive abilities can lead to problems when conducting digitized assessments(Silverberg et al., 2011) cannot be confirmed by the study.

The second hypothesis that the electronically recorded execution time corresponds to the manually measured execution time can also be confirmed. This is in accordance with a study by Dahmen et al. equally based a digitalized version of the TMT (Dahmen, Cook, Fellows, & Schmitter-Edgecombe, 2017). Within the study, the authors showed that the predicted digital TMT scores correlate significantly with clinical digital test scores. In the study by Dahmen et al., the authors also investigated several additional features (besides time to completion and number of errors) such as timing features (e.g., average pause duration and average lift duration) and mobility features (e.g., number of pauses, number of lifts, and pressure). The results showed that the inclusion of all parameters (mobility and timing) does not provide the best prediction for test results. Furthermore, in the present study, it could be shown that not all additionally recorded parameters correlate with the test result. Only the number of pen lifts and the number of errors showed a significant influence on the duration of the test in TMT B.

A study by Bracken et al. postulates that new standard values must be generated for the application of digital or computerized test procedures, which means a considerable effort (Bracken et al., 2019). This seems not necessary for the use of the digital pen technology, because the method of execution is almost identical.

### Limitations

Within the presented study, we investigated only a small homogenous sample, including well-educated, healthy participants. Although participants with severe cognitive, auditory, and visual impairments were excluded in the study, there may be differences in the cognitive and sensory abilities of the subjects. These abilities were not considered in this study. It is possible that our findings will not apply to more heterogenous samples, especially for participants with severe cognitive disorders. Due to the healthy sample, almost all test persons scored average or above average in the TMT, so we could not determine whether the significant differences in the mean values would misclassify some patients. Furthermore, the number of errors made were not recorded by the study personal, so this could not be compared between the manual and the electronical results. Problems that can occur when using technical systems, such as the failure of the technology, the need for recharging, connection problems or the higher price (in comparison to a pencil), were not considered in the present study.

## Conclusion

A digital pen can be used to perform the Trail Making Test, as it has been shown that there are no differences in results due to the type of pen used. The parameters recorded in the paper-based version (time, errors) are easily measured digitally, reducing measurement errors and minimizing the influence of the rater. To conclude, the advantages of digitalized cognitive assessment can be used without suffering the disadvantages. The evaluation of additional parameters did not correlate with the test results in the presented study and should be considered with caution when evaluating the results.
